# Neuropsychiatric manifestations of primary hyperparathyroidism

**DOI:** 10.1007/s00508-025-02688-3

**Published:** 2026-01-08

**Authors:** Marco Mairinger, Godber Mathis Godbersen, Siegfried Kasper

**Affiliations:** 1 Department of Psychiatry and Psychotherapy, Medical University of Vienna, Vienna, Austria; 2 Comprehensive Center for Clinical Neurosciences and Mental Health, Medical University of Vienna, Vienna, Austria; 3 Center for Brain Research, Department of Molecular Neuroscience, Medical University of Vienna, Spitalgasse 4, 1090 Vienna, Austria

**Keywords:** Calcium, Behavioral endocrinology, Depression, Cognitive dysfunction, Parathyroidectomy

## Abstract

Neuropsychiatric manifestations occur in about 25% of patients with primary hyperparathyroidism (PHPT). Symptoms can range from depression, anxiety, fatigue or cognitive dysfunction which are commonly observed to more seldomly revealed states of mania, delirium or psychosis which warrant psychiatric intervention. The underlying pathophysiology is likely multifactorial, potentially explained by elevated parathormone and hypercalcemia, with subsequent direct and indirect effects on monoamine neurotransmission and neuroinflammation via monoamine oxidase, tyrosine hydroxylase, sodium-potassium adenosine triphosphatase transporter and interleukin‑6.

This review aims to (1) give an overview of the hypothesized pathophysiologic understanding regarding neuropsychiatric manifestations, (2) to summarize the most common neuropsychiatric symptoms and (3) to equip clinicians with recommendations for evidence-based tools to detect neuropsychiatric symptoms effectively.

Psychometric questionnaires depicting psychiatric symptom burden across PHPT research are highlighted. Cut-off values for psychiatric screening purposes and hypothesized cut-off values in PHPT research to indicate parathyroidectomy are provided. A practical approach on how screening for neuropsychiatric symptoms in PHPT might be implemented in routine clinical practice is outlined.

Parathyroidectomy is recognized to alleviate neuropsychiatric symptoms in PHPT, with increasing evidence showing persistent improvements in symptoms of depression, anxiety, fatigue and cognitive dysfunction. Clinical practice guidelines still diverge on whether neuropsychiatric manifestations in PHPT warrant parathyroid surgery. A symptom-based treatment approach is recommended alongside evaluating surgical intervention.

## Introduction

About 25% of patients with primary hyperparathyroidism (PHPT) suffer from a wide range of neuropsychiatric symptoms [1, 2]. Documented neuropsychiatric manifestations range for more commonly depressive and anxiety-related symptoms, fatigue, cognitive impairment in memory and concentration to sleep disturbances [2–4]. More seldomly observed symptoms but requiring urgent psychiatric evaluation and treatment, are acute or prolonged states of delirium, catatonia, psychosis and mania [5–9]. Associations of PHPT and eating disorders are very scarce but observed in a handful of case-reports [6, 10]. Currently, there are no psychiatric disorders known to be associated with an increased risk for PHPT. According to the International Classification of Diseases 10th Revision (ICD-10), psychiatric disorders tend to be a diagnosis by exclusion, when symptoms are better explained by other causes [11].

This review focuses primarily on neuropsychiatric manifestations of PHPT and aims to (1) give an overview of the current pathophysiologic understanding of neuropsychiatric manifestations in PHPT, (2) summarize the most common neuropsychiatric symptoms and (3) equip clinicians with recommendations for evidence-based tools to detect neuropsychiatric symptoms effectively and efficiently.

## Pathophysiology of neuropsychiatric manifestations in PHPT

The first cohesive hypothetical model aiming to explain shared mechanisms in PHPT, biochemically characterized by elevated parathyroid hormone (PTH) and calcium levels, leading to the multitude of diverging neuropsychiatric symptoms clinically observed, is provided by Serdenes et al. [12], bringing together proposed mechanisms of isolated neuropsychiatric symptoms throughout the literature and basic science knowledge.

Current research summarized by Serdenes et al. [12] suggests that effects of PHPT either increase or decrease monoamine neurotransmission. They theorize that changes in monoamine neurotransmission may be dependent on an individual’s unique neuroanatomical structure and predisposition, potentially leading to differences in emerging neuropsychiatric symptoms, which might explain the wide range of observed neuropsychiatric manifestations in PHPT.

Serdenes et al. [12] highlighted six total variables likely to be involved in the underlying pathophysiology: tyrosine hydroxylase, PTH, interleukin‑6 (IL-6), monoamine oxidase (MAO), calcium and the sodium-potassium adenosine triphosphatase (Na/K-ATPase) Transporter.

The mechanisms observed to increase PHPT-related monoamine neurotransmission, laid out by Serdenes et al. [12, 13] are reduced MAO function, higher levels of synaptic calcium and decreased function of Na/K-ATPase. The MAO activity seems to be impaired in hypercalcemic states leading to reduced affinity of substrates, subsequently leading to increased neurotransmission of monoamines, such as dopamine, serotonin and norepinephrine [12, 14]. The authors inform that hypercalcemia has additionally been shown to cause changes in presynaptic calcium influx, followed by synaptic vesicle release, hypothesized to increase monoamine neurotransmission [5, 12]. Gheorge et al. [15–17] presented contrasting evidence of MAO‑A activity increasing, with higher cerebral levels of calcium suggesting a decrease rather than an increase in monoamine neurotransmission. The PTH seems to increase synaptic cleft norepinephrine concentrations via inhibition of synaptic norepinephrine reuptake mediated by Na/K-ATPase through incompletely elucidated mechanisms, as Serdenes et al. [12, 13] described.

Mechanisms observed to decrease PHPT-related monoamine neurotransmission, summarized by Serdenes et al. [12], are reduced function of tyrosine hydroxylase, increased cerebral levels of PTH and increased levels of IL‑6. They lay out that hypercalcemia has been associated with reduced tyrosine hydroxylase function, resulting in decreased levels of dopamine and norepinephrine [12, 14]. The PTH has been observed to cross the blood-brain barrier with conflicting evidence and is associated with states of depression, independent of hypercalcemia and calciferol [12, 15, 18]. A recent review summarized work that indicates expression of PTH1R and PTH2R in various human brain regions (hippocampus, hypothalamus, amygdala, cerebellum), supporting neuromodulating involvement and direct effects of PTH [17, 19–27]. The PTH2 receptors located in the limbic system were described by Serdenes et al. [12, 28] to be a potential pathway affecting psychiatric symptoms. Interestingly, recent studies involving animal models demonstrated PTH binding to PTH2 receptors to be associated with states of anxiety, stress and emotional regulation, strengthening potential involvement [29]. As laid out in a review by Gheorghe et al. [15], PTH binds to the ligand tuberoinfundibular peptide of 39 residues (TIP39) with higher affinity than PTH2 receptors, with TIP39 subsequently binding to PTH2 receptors, which was associated with anxiety, stress and nociception via modulation of the hypothalamic-pituitary-adrenal axis in animal models [17, 27, 29, 30]. Furthermore, experimental research demonstrated that PTH activates neurons in the circumventricular subfornical organ in mice models, suggesting a central signaling mechanism for neuromodulation without the need to pass the blood-brain barrier [31]. Serdenes et al. [12] mention that changes in monoamine neurotransmission have been observed through cytokines, as in IL‑6, which appear to be increased by PTH and associated with depressive symptoms [32, 33]. Increasing evidence suggests a connection of inflammatory processes and neuropsychiatric disorders [34]. Interestingly, a recent systematic review identified IL‑6 and tumor necrosis factor (TNF)-alpha levels to be elevated in PHPT patients, normalizing after parathyroidectomy [35].

The combination of PTH-mediated effects increasing monoamine neurotransmission might result in presentations of mania or psychosis as Serdenes et al. [12] suggest, with decreased monoamine neurotransmission potentially resulting in symptoms of depression, anxiety or cognitive dysfunction. Delirium might be influenced not only by monoamine neurotransmission but also through severe hypercalcemia, as serum-calcium concentrations above 13.5 mg/dL can lead to stupor [12, 36]. Seldomly observed manifestations of eating disorders or catatonia may be explained by complex changes in monoamine neurotransmission [10, 12, 37]. Concentration-dependent and second messenger effects of calcium are plausible but remain to be elucidated for PHPT-related neuropsychiatric manifestations [1, 12, 38, 39].

To summarize, the underlying pathophysiology of neuropsychiatric symptoms in PHPT is likely multifactorial, with increasing and contrasting evidence implicating direct effects of PTH, inflammatory states brought on by PHPT, alterations in monoamine neurotransmission and hypercalcemia.

## Depression and anxiety-related symptoms

A recent review by Singh et al. [4] summarized the existing literature for depressive symptoms in PHPT. In essence, depressive symptoms are highly prevalent but not easily distinguishable from major depressive disorder (MDD) as most studies to date [4] used different rating scales to quantify symptoms. Despite the high prevalence of MDD in the general population, MDD is the most prevalent co-occurring psychiatric diagnosis in PHPT patients and depressive symptoms depict the most commonly reported neuropsychiatric manifestation of PHPT [4]. Promising evidence for a potential causal relationship of depressive symptoms or MDD in PHPT are trials incorporating standardized rating scales and ICD-10 diagnostic criteria measuring symptom load before and after parathyroidectomy at subsequent timepoints with implemented control groups, as synthesized by Singh et al. [4, 40, 41].

Depressive symptoms, quantified by a Beck Depression Inventory II (BDI-II) score, are significantly higher in PHPT patients compared to control groups and show a statistically significant decrease in BDI-II scores and therefore depressive symptom severity at under 1 month and 6 months postoperatively, as revealed in a recent meta-analysis of 9 cohorts and 842 participants, whereas only 2 cohorts featured subsequent control groups [42]. The authors proposed a BDI-II score of ≥ 14, a cut-off score mostly relating to a mild depressive episode, as a potential threshold for surgical intervention [42].

To add on, a recent meta-analysis investigating the course of psychiatric symptom load before and after parathyroidectomy via Patient Health Questionnaire‑9 (PHQ-9) and Generalized Anxiety Disorder‑7 (GAD-7) scores in 1105 participants across 6 cohorts revealed higher baseline depressive and anxiety symptom load compared to controls [43]. With a surgical intervention, the percentage of patients with a PHQ‑9 score of ≥ 10, indicating clinically significant depressive symptoms, decreased dramatically [43]. Furthermore, improvements in anxiety symptoms and suicidal ideation were observed. The authors propose a PHQ‑9 score of ≥ 10 as a potential cut-off value for parathyroidectomy [43]. The authors abstained from proposing a cut-off value for anxiety-related symptoms despite postoperative symptom improvements because of limited data points.

This is in line with another meta-analysis investigating improvements of depressive symptoms in PHPT pre-parathyroidectomy and post-parathyroidectomy [44]. Across 26 studies and 2365 participants, significant improvements in depressive symptoms measured by BDI-II and PHQ‑9 were observed up to 3 months (effect estimate = 0.57; 95% confidence interval, CI, 0.33–0.81) and beyond 12 months (effect estimate = 0.46; 95% CI, 0.21–0.71) postoperatively, whereas no consistent significant improvement was found between the timeframe of 3–12 months (effect estimate = 0.68; 95% CI −0.32–1.69) postoperatively [44].

Interestingly, an article evaluating the risk of neuropsychiatric disorder onset in PHPT comparing parathyroidectomy to nonoperative management revealed no reduction in the hazard ratio for suicidal ideation, anxiety-disorders or mood disorders in a cohort of 3278 patients, where institutional patient records were retrospectively analyzed with a median follow-up time of 4.2 years in the USA [45].

Further research is needed to validate existing psychometric symptom scales for PHPT patients and investigate proposed cut-off values, which might differ in PHPT patients compared to the general population regarding neuropsychiatric symptom severity.

## Fatigue

Fatigue is a commonly reported symptom in PHPT, impacting the quality of life. In a sample of 2197 PHPT patients, 63% presented with fatigue [46]. Other authors [47] propose a prevalence of up to 98% in a cohort of 132 PHPT patients. A significant reduction in subjectively reported fatigue was found following parathyroidectomy, compared to baseline [46, 47]. Specific self-rating measures for fatigue are not implemented to the best of our knowledge in PHPT research yet, as existing research relies primarily on reports of fatigue in a yes/no fashion or on a 5-point Likert scale during clinical examination. As the definition of fatigue is generally vague, it might be used by patients and providers alike as an umbrella term, potentially encompassing a multitude of symptoms regarding anhedonia, adynamia, loss of energy, general weakness or even sleep disturbances, showing the need for more detailed exploration regarding neuropsychiatric symptoms to reach an understanding of what “fatigue” means to the individual patient. Fatigue might occur as a standalone neuropsychiatric symptom or in conjunction with a symptom cluster better conceptualized by the well-established term depressive episode. According to ICD-10 diagnostic criteria, a depressive episode features three main symptoms, depressed mood, loss of joy or interest (anhedonia) and reduction in energy with resulting decrease in activity as well as minor symptoms including feelings of reduced self-worth, feelings of guilt/worthlessness, suicidality and/or self-harming behavior, difficulties concentrating, psychomotor agitation or retardation, increase or decrease in appetite and hypersomnia or insomnia [11]. The diagnostic criteria are met when at least 2 major symptoms are present over a period of 2 weeks. Minor symptoms help in determining severity of a minor (0–2 minor symptoms), moderate (3 minor symptoms) or severe (at least 4 minor symptoms) depressive episode. If such distinct episodes occur at least twice over an individual’s lifetime, the criteria for MDD are met [11].

Subsequently, asking about feelings of fatigue is of importance during clinical examination to gauge the subjective burden in daily life and might function as an opener question to further examine distinct neuropsychiatric symptoms.

In future studies, depicting reported fatigue with validated self-rating assessment scales for chronic diseases, such as the fatigue assessment scale, in conjunction with already established depressive symptom rating scales might be interesting to determine whether fatigue should be measured as a standalone symptom or is sufficiently depicted within the depressive symptom cluster [48].

To utilize existing research subgroup analyses in large cohorts using the PHQ‑9 or BDI-II can be performed for items depicting reported fatigue and where available, comparing subsequent timepoints before and after parathyroidectomy for severity of fatigue over time [42, 43]. This may be useful to determine overlap and potential distinction of MDD, depressive symptoms and fatigue.

## Neuromuscular manifestations

Objective loss of muscle strength is observed in PHPT patients and might provide further diagnostic information when assessed during clinical examination, depicting the ability to participate in activities of daily life and identify individuals at risk of frailty [49, 50]. A recent study [51] provided preliminary evidence for increases in objective muscle strength without changes in physical activity levels following parathyroidectomy as well as increased gene expression with enrichment in physical activity-induced pathways in transcriptomic analysis.

In addition to reduced muscle strength neuromuscular symptoms encompassing polyneuropathy, muscle cramps and paresthesia have been reported in observational studies, as described in a review by El-Hajj Fuleihan et al. [2] and might be linked with increased PTH levels. The authors [2] emphasize, however, that current data remain insufficient to infer a direct association of such symptoms and PHPT. A detailed overview regarding the relationship of PTH, calcium, bone-muscle and nerve-muscle interactions was recently reviewed by Nguyen et al. [52].

To the best of our knowledge, no observational studies, randomized controlled trials, reviews or meta-analyses have reported an association of PHPT and relevant occurrence of Parkinsonism-associated-tremors and seizures to date, despite observations in case reports and subsequent symptom improvement following parathyroidectomy [53–55]. This may be due to the clinical advancements in PHPT detection and subsequently earlier interventions, preventing cases of long-persisting severe hypercalcemia and therefore onset of some symptom clusters [56].

## Cognitive function and dysfunction

A recent review by Singh et al. [4] managed to expand on the potential effect of PHPT on cognition as elaborated previously in reviews by Lourida et al. [57] and Jiang et al. [58], by incorporating relevant findings up to the year 2024. No independent effect of PTH levels and cognitive decline across a 20-year timeframe was observed in a sample of 12,964 individuals [59]. Current knowledge suggests an association of increased PTH levels and the development of vascular dementia, whereas no changes in risk are observed for Alzheimer disease [4, 60]. As distilled in more detail by Singh et al. [4], reduced cognitive function, memory or concentration, inferred by reduced scores of the mini-mental state exam (MMSE) and reduced scores in neuropsychiatric test batteries are observed in PHPT patients across cohorts, with mixed results in older trials regarding cognitive improvement after parathyroidectomy. Nonetheless, due to major limitations and heterogeneity in assessment scales, follow-up-time, varying implementation of control groups and the inconsistency of incorporating confounding variables, more prospective research is needed to infer a potential causal relationship. As highlighted by Singh et al. [4], promising results of more recent studies demonstrate improvements of cognition in MMSE at 2 and 6 months post-parathyroidectomy.

Objective improvements of cognitive function were found in a sample of 59 patients with PHPT before and after parathyroidectomy, in comparison to the general population. Neurocognition was measured by a clinically validated psychological test battery, BrainCheck, with 44.1% of patients demonstrating objective neurocognitive dysfunction before parathyroidectomy and only 22% of patients postoperatively [61].

As mentioned before a retrospective analysis of 3728 PHPT patient records in a North America based cohort evaluated the risk of developing neuropsychiatric disorders following parathyroidectomy compared to nonoperative management [45]. The cohort receiving surgical interventions demonstrated a lower hazard ratio (HR) for somnolence (HR: 0.45, 95% CI: 0.23–0.9), new onset cognitive impairment (HR: 0.65, 95% CI: 0.47–0.91) and schizophrenia (HR: 0.08, 95% CI: 0.01–0.6) compared to the nonoperative management group [45]. A reduced need for psychiatric inpatient care was observed in the surgical intervention group (0.3% vs. 1.8%, *p* < 0.001) [45].

A recent prospective observational study evaluated the effect of calcimimetic therapy with cinacalcet at baseline and follow-up after 1 month and subsequently 6 months after parathyroidectomy across cognitive performance metrics, psychiatric symptom load and muscle strength [62]. Cognitive dysfunction, as measured by the Montreal cognitive assessment (MoCA) decreased after calcimimetic therapy [62]. Improvements in MoCA and baseline calcium blood-levels were found to be independent predictors of favorable surgery outcomes [62].

## Implementation of psychometric assessment in clinical practice

In clinical practice, a time-efficient and cost-efficient way to incorporate these findings might be by implementing screening questionnaires to quantify neuropsychiatric symptom prevalence and severity in a standardized manner during routine assessments, when PHPT is clinically suspected or persistent hypercalcemia is observed in a primary care setting, in surgical outpatient centers managing PHPT, during surgical planning and postoperative care.

Practically, one screening questionnaire per symptom cluster (depression, anxiety, cognitive dysfunction) would help inform clinicians about a multitude of typical manifestations. Self-rating questionnaires for depressive symptoms include the PHQ‑9 and BDI-II, with the Hospital Anxiety and Depression Scale (HADS) simultaneously assessing symptoms of anxiety and the GAD‑7 evaluating anxiety exclusively [63–66]. Screening for cognitive dysfunction and signs of dementia can be done with the MMSE or MoCA [67, 68]. Both are external rating questionnaires and can be administered by trained physicians, psychologists, nurses, medical or nursing students, with the MoCA being more sensitive in detecting early stages of mild cognitive impairment, where the MMSE often depicts normal scores [67, 68]. If staffing is an issue, most self-rating questionnaires can be administered digitally via tablets or smartphones with automatic scoring. Just recently, MoCA Cognition released an app, XpressO, featuring a digital self-rating alternative with comparable sensitivity and specificity to the MoCA, making a fully digital implementation feasible [69]. The waiting time during routine visits could be utilized to administer rating scales.

A tailored psychological test battery (e.g., BrainCheck), specifically constructed and validated for PHPT patients, performed by a trained psychologist or psychiatrist would be ideal but does not always appear to be feasible in routine clinical practice for screening purposes. This may be better reserved for a subgroup of patients with high clinical suspicion of neuropsychiatric manifestations, either depicted during psychometric questionnaire assessment or during clinical interviews.

In Table 1, relevant psychometric scales suitable for general psychiatric screening purposes, which are already widely used in PHPT research, are provided alongside depicted symptom clusters, cut-off values in the general population and proposed cut-off values in PHPT research to guide indication of parathyroidectomy.

**Table 1:** Overview of neuropsychiatric symptom clusters in PHPT and respective screening questionnaires to guide clinicians in detecting neuropsychiatric manifestations alongside cut-off values indicating clinically relevant psychiatric burden. Additionally, proposed cut-off values of PHPT research are presented, which may guide indications for parathyroidectomy. [42, 43, 63–68]

	Typical manifestations	Rating scales	Cut-off values general population	Usage in PHPT research
Depressive symptoms	Sadness/low mood Loss of energy Changes in activity Anhedonia Sleep disturbances Fatigue Changes in appetite Feelings of guilt	PHQ‑9	≥ 10 mild depression [63] → psychiatric referral item 9, suicidality ≥ 1 → warrants immediate suicidal risk assessment [63]	Score ≥ 10 → proposed cut-off value for parathyroidectomy indications in research [43]
		BDI-II	≥ 14 mild depressive episode [64] → psychiatric referral	Score ≥ 14 → proposed cut-off value for parathyroidectomy indications in research [42]
		HADS	≥ 8 depression subscale [65]—clinically relevant depressive symptoms → psychiatric evaluation ≥ 8 anxiety subscale [65]—clinically relevant anxiety symptoms → psychiatric evaluation	Proposed as a screening-tool for depressive and anxiety symptoms
Anxiety symptoms	Generalized worry Muscle tension/pain Excessive sweating Trembling Palpitations/tachycardia Paresthesia/Hypoesthesia	GAD‑7	≥ 8 indicates clinically relevant anxiety symptoms [66] → psychiatric evaluation	Proposed screening tool for anxiety symptoms
		HADS	≥ 8 anxiety subscale [65]—clinically relevant anxiety symptoms → psychiatric evaluation ≥ 8 depression subscale [65]—clinically relevant depressive symptoms → psychiatric evaluation	Proposed as a screening tool for depressive and anxiety symptoms
Cognitive function	Memory impairment Concentration difficulties Slowed processing speed	MoCA	≤ 25 indicates mild cognitive impairment [68], ≤ 18 indicates dementia [68] → neuropsychiatric referral	Proposed clinician administered screening tool to detect cognitive dysfunction
		MMSE	≤ 24 indicates dementia [67] → neuropsychiatric referral	Proposed clinician administered screening tools to detect cognitive dysfunction

*PHPT* primary hyperparathyroidism, *PHQ‑9* patient health questionnaire‑9, *BDI-II* Beck depression inventory-II, *HADS* hospital anxiety and depression scale, *GAD‑7* generalized anxiety disorder‑7, *MoCA* Montreal cognitive assessment, *MMSE* mini-mental state exam

## Outlook and treatment of neuropsychiatric manifestations

In the 2021 German and Austrian clinical practice guidelines [70] parathyroidectomy is recommended for patients with typical psychiatric and neurocognitive symptoms in the hope to improve symptoms. Conversely, the 2022 international clinical practice guidelines [71] determined the evidence to be insufficient to recommend parathyroidectomy with the expectation of neuropsychiatric symptom remission.

More observational and randomized controlled trials comparing either depressive symptom severity, anxiety-related symptom severity and cognitive dysfunction, demonstrated a significant reduction of symptoms following parathyroidectomy across various psychometric rating scales and quality of life [44, 61]. Subsequently, this strengthens the argument to recommend treatment with parathyroidectomy in moderate to severe neuropsychiatric manifestations of PHPT and otherwise asymptomatic cases and cautiously expect neuropsychiatric symptom improvement in a subset of patients.

Nevertheless, in a subset of patients, neuropsychiatric symptoms tend to persist after parathyroidectomy, which underscores the importance of incorporating screening tools and interview questions about neuropsychiatric symptoms regularly throughout the treatment period, to identify patients who may benefit from operative intervention and systematically evaluate the effects of parathyroidectomy on neuropsychiatric symptoms.

Furthermore, individuals presenting with signs of neuropsychiatric disorders, either caused by PHPT or detected as a comorbidity, can be identified and guided towards psychiatric evaluation and symptom-based psychopharmacological treatment. Vice versa, psychiatrists may evaluate reported symptoms of depression, anxiety, cognitive dysfunction, muscle weakness and states of delirium, mania or psychosis in psychiatric patients with increased awareness of current serum calcium levels, as underlying hypercalcemia and PHPT may be a causal factor in some patients. This should especially be considered in psychiatric patients with insufficient psychopharmacological treatment response. This highlights the need to further advance interdisciplinary approaches in detecting and managing PHPT.
